# Flash glucose monitoring system in special situations

**DOI:** 10.20945/2359-3997000000479

**Published:** 2022-06-02

**Authors:** Fernanda Augustini Rigon, Marcelo Fernando Ronsoni, André Gustavo Daher Vianna, Leonardo de Lucca Schiavon, Alexandre Hohl, Simone van de Sande-Lee

**Affiliations:** 1 Universidade Federal de Santa Catarina Programa de Pós-graduação em Ciências Médicas Florianópolis SC Brasil Programa de Pós-graduação em Ciências Médicas, Universidade Federal de Santa Catarina, Florianópolis, SC, Brasil; 2 Universidade Federal de Santa Catarina Departamento de Clínica Médica Florianópolis SC Brasil Departamento de Clínica Médica, Universidade Federal de Santa Catarina, Florianópolis, SC, Brasil; 3 Hospital Nossa Senhora das Graças Departamento de Doenças Endócrinas Centro de Diabetes de Curitiba Curitiba PR Brasil Centro de Diabetes de Curitiba, Departamento de Doenças Endócrinas, Hospital Nossa Senhora das Graças, Curitiba, PR, Brasil

**Keywords:** Diabetes mellitus, blood glucose self-monitoring, liver cirrhosis, renal dialysis, pregnancy

## Abstract

The management of diabetes mellitus (DM) requires maintaining glycemic control, and patients must keep their blood glucose levels close to the normal range to reduce the risk of microvascular complications and cardiovascular events. While glycated hemoglobin (A1C) is currently the primary measure for glucose management and a key marker for long-term complications, it does not provide information on acute glycemic excursions and overall glycemic variability. These limitations may even be higher in some special situations, thereby compromising A1C accuracy, especially when wider glycemic variability is expected and/or when the glycemic goal is more stringent. To attain adequate glycemic control, continuous glucose monitoring (CGM) is more useful than self-monitoring of blood glucose (SMBG), as it is more convenient and provides a greater amount of data. Flash Glucose Monitoring (isCGM /FGM) is a widely accepted option of CGM for measuring interstitial glucose levels in individuals with DM. However, its application under special conditions, such as pregnancy, patients on hemodialysis, patients with cirrhosis, during hospitalization in the intensive care unit and during physical exercise has not yet been fully validated. This review addresses some of these specific situations in which hypoglycemia should be avoided, or in pregnancy, where strict glycemic control is essential, and the application of isCGM/FGM could alleviate the shortcomings associated with poor glucose control or high glycemic variability, thereby contributing to high-quality care.

## INTRODUCTION

The management of diabetes mellitus (DM) primarily requires effective glycemic control since reductions of glycemic variability can minimize the micro and macrovascular complications associated with the disease (1). Glycated hemoglobin (A1c) remains the reference test to monitor the glucose level of patients with DM and estimate the average blood glucose levels of two to three months (2). However, this measure fails to consider fluctuations in blood glucose levels during the day and fails to detect acute events of hypoglycemia or postprandial hyperglycemia. Moreover, the method is unreliable in measuring blood glucose levels during anemia, in pregnant patients, and in many other situations (3,4).

Self-monitoring of blood glucose (SMBG), which is usually performed with finger-prick blood samples, though useful in the management of the disease, does not provide continuous data because the values are not obtained in a timely manner, depending upon the patient's decision to test (5,6). Various factors, such as pain and inconvenience, lead to lower evaluations frequency and difficulties in diagnosing nocturnal hypoglycemia (7,8).

## CONTINUOUS GLUCOSE MONITORING (CGM)

The FreeStyle Libre™ Flash Glucose Monitoring System (isCGM/FGM)(Abbott Diabetes Care, Alameda, CA) is an interstitial continuous glucose monitoring (CGM) system that uses a glucose sensor inserted into the skin over the triceps and a portable reader, which people can use to scan their blood glucose level. The sensor automatically measures glucose every minute, storing the values in 15-minutes intervals (9). It is factory-calibrated and requires no fingerstick for calibration. However, a confirmation with capillary blood glucose (CBG) is recommended in the event of sensor-reported hypoglycemia, during rapidly changing glucose concentrations, in the first 24 hours after sensor insertion, and when no correspondence is identified between symptoms and interstitial glucose (10).

Thus, it is critical to understand that each physiological compartment follows different dynamics, and several factors cause concentration disparities between blood and interstitial fluid (ISF) glucose levels. These factors include the distribution of glucose between blood vessels and subcutaneous tissue, glucose permeability, blood flow, and the release of pancreatic hormones such as insulin and glucagon. Moreover, a physiological time lag between blood and ISF glucose levels is remarked. When such levels are changing rapidly, their measurements differ considerably (5,6).

**Figure 1:**
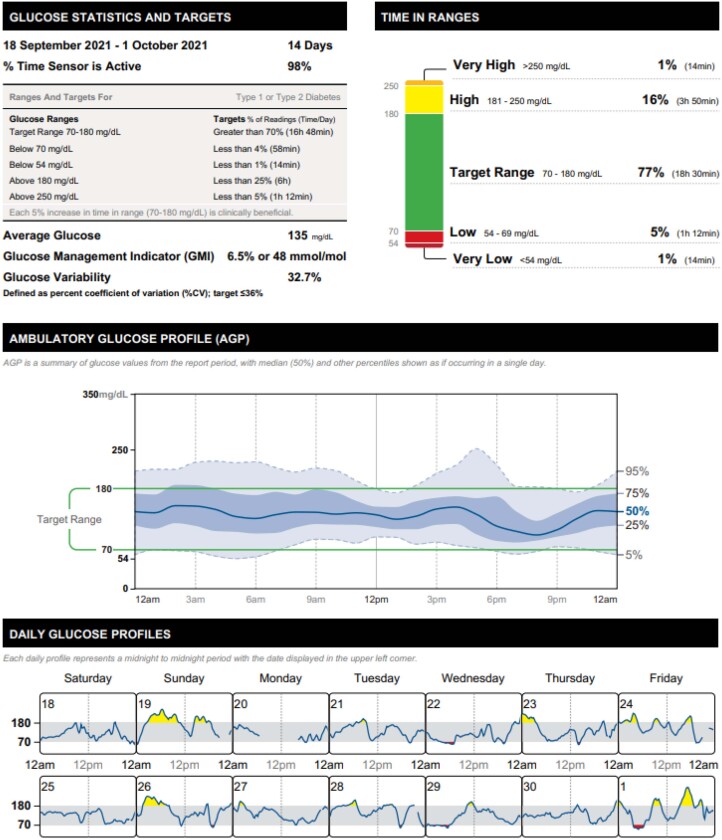
Example of an ambulatory glucose profile report, a standardized single-page report developed by the International Diabetes Center and adopted by most of the CGM device manufacturers, including 14 days of data. The middle graph shows the median glucose over 24 hours and its variation (5^th^, 25^th^, 75^th^, and 95^th^ percentile), while the bottom graph shows daily glucose profiles. The stacked bar graph at the top right corner displays the percentage of time spent within, below, and above the target range. The additional table at the top left corner describes glucose statistics and targets.

Time in range (TIR) is the most relevant parameter to assess glucose control, which indicates the percentage of time that a person spends with their interstitial glucose levels in the target range, usually defined between 70 and 180 mg/dL (Figure 1). Moreover, it is possible to evaluate the average glucose, glucose management index, glucose variability, time in hypoglycemia (time below range, TBR) and hyperglycemia (time above range), and the number of reads that the user performed per day (11).

The primary goal for effective and safe glucose control for an individual with DM is to increase the TIR to over 70% while reducing the TBR to less than 4, thereby including the time in level 2 hypoglycemia (below 54 mg/dL) to less than 1%. Thus, CGM-based glycemic targets can be personalized to address specific needs of special diabetes populations (12).

The association between TIR and microvascular complications was reported in previous studies, where individuals with advanced diabetic retinopathy and chronic kidney disease spent less time on target, while the highest TIR was associated with lower frequency of these complications (13,14). In randomized clinical trials, a TIR of 70% and 50% corresponded to an A1c of approximately 7% and 8%, respectively. Meanwhile, an 8% to 10% increase in TIR was associated with a 0.5% decrease in A1c (15,16).

Recently, the new version of FreeStyle Libre 2TM has been launched in some countries; BluetoothTM technology has been integrated into the system, where an optional alarm is available to alert patients in case of high and low glucose levels (17). FreeStyle Libre 3TM is already approved in Europe, and it offers similar features to that of the previous version plus some additional features, such as continuous real-time glucose readings that automatically delivers glucose level to a smartphone every minute through a sensor. The FreeStyle Libre ProTM is a device that uses the same system, but it is blind to the patient. Healthcare professionals applied the sensor, scanned with a reader after 14 days, and downloaded the results stored in the sensor.

Bailey and cols. compared capillary and venous glucose with ISF glucose using the isCGM/FGM first-generation system in 2015. The overall mean absolute relative difference (MARD) was 11.4%. In zones A and B of the consensus error grid, the percentage of sensor results was 99.7%. Sensor accuracy was not affected by factors such as body mass index, age, type of diabetes, clinical site, insulin administration, or A1c (9).

Another study evaluated FreeStyle Libre 2TM performance to compare plasma venous blood glucose. The updated system demonstrated improved analytical accuracy performance across the dynamic range during the 14-day sensor wear period, with a 9.2% overall MARD and 9.7% for the pediatric population (18).

In some special situations, frequent glucose monitoring is crucial to avoid complications and SMBG is a barrier to optimal glucose control. Recent studies show a positive correlation between the frequency of blood glucose assessments and glycemic control (9,17,19–21).

The main advantages of the isCGM/FGM are its convenience for patients and the greater amount of data available from SMBG (22). When comparing isCGM/FGM with SMBG, TBR was reduced by 38% in patients with type 1 diabetes mellitus (T1DM) in the IMPACT study and 43% in individuals with type 2 diabetes mellitus (T2DM) in the REPLACE study. In both studies, A1c was similar between groups, revealing a significant improvement in quality of life in groups using isCGM/FGM (17,19).

However, some shortcomings of isCGM/FGM include the lower accuracy for low glucose values and lack of a sound alarm to alert patients about hypoglycemia and hyperglycemia. However, this issue has been recently solved with the launching of a new version. Furthermore, the device offers no option to connect to an insulin pump, requiring an exclusive reader or an application in a compatible smartphone (10).

The present review discusses the benefits of isCGM/FGM in diagnosing poor glucose control or high glycemic variability, thereby enhancing effective decisions regarding the treatment of diabetes. In addition, the study discusses specific situations in which hypoglycemia is avoided or strict glycemic control is vital, in which the current metrics are inadequate (not mentioned). Many studies evaluated Libre 1 sensors.

In cases where Libre 2 or Libre Pro sensors were used, this information was included in the text.

## PREGNANCY

The relationship between hyperglycemia during pregnancy and adverse outcomes for mother and fetus is well established in the literature (23). These complications include spontaneous abortion, preterm births, large for gestational age (LGA), and neonatal hypoglycemia (23). In women with T1DM and T2DM, tight glucose control is vital from the pre-pregnancy to the post-pregnancy period to reduce fetal and maternal risks (24,25).

However, A1c is unreliable for assessing glucose control during pregnancy owing to physiological changes that compromise its measurement, which include an increase in production of red blood cells, increase of half-life of cells, decrease in the affinity of hemoglobin to glucose, and iron deficiency (26,27).

The pregnancy affects glycemic control and the management of diabetes since insulin resistance decreases in the first trimester and increases in the second half of pregnancy (28). The risk of severe hypoglycemia is a barrier to maintaining strict glycemic control. Hypoglycemia can be five times more frequent at the beginning of pregnancy than in the pre-gestational period in women with T1DM (29).

Women with gestational diabetes mellitus (GDM) face a particular challenge. Thus, they must understand disease management, which includes techniques to monitor and control blood glucose, the effects of diet, the effects of physical exercise, and medication on their glucose. They must optimize their glucose control within the pregnancy time frame to minimize complications (30).

SMBG should be performed at least four to eight times a day to reach the rigorous glycemic targets in pregnancy. Moreover, frequent tests are critical for the patient on intensive insulin treatment; however, conducted less frequently when on diet therapy or a basal insulin regimen (31). Nonetheless, SMBG may fail to capture transient glucose excursions, as the values are assessed intermittently, hindering proper control (30).

Continuous glucose monitoring provides additional data without discomfort, which has been attributed to SMBG. The CONCEPTT (Continuous Glucose Monitoring in Women With Type 1 Diabetes in Pregnancy Trial) was a multicenter randomized controlled trial involving 215 pregnant women with T1DM. The study reported improved neonatal outcomes with a 5% increase in TIR among the real-time CGM (rtCGM) group (32). Scott and cols. analyzed the data from the CONCEPTT study and revealed that the rt-CGM reduced fetal glucose exposure, especially during the day, suggesting that it was easier to monitor the effect of feeding with rtCGM. The strategy assists in preventing and managing fluctuations in glucose levels (33).

Kristensen and cols. observed that a 5% to 7% increase in TIR during the second and third trimesters of pregnancy in women with T1DM was associated with a lower risk of LGA newborns and with a decrease in neonatal complications (34). Murphy and cols. reported a positive association between glycemic control and the outcomes of pregnant women with T1DM and T2DM (35). The same benefits were observed in a recent systematic review that focused on the effect of CGM on GDM (36).

Despite the positive outcomes presented by the recent CONCEPTT study, over 80% of the women experienced frustrations with CGM, such as connectivity issues, alarms, and calibration errors. Moreover, 48% of participants experienced skin reactions, such as bleeding, erythema, and discomfort following sensor use (32). Other reported barriers to the use of CGM include technical challenges, calibration, skin irritation, frequent alarms (especially during sleep), accuracy, and cost, which discourage its use (37,38).

Thus, isCGM/FGM is more acceptable by pregnant women than other CGM devices owing to its simplicity of handling the device, the absence of alarms, or the possibility of adjusting personalized glucose limits (30). Moreover, isCGM/FGM was recently approved for pregnant women, as it targets 70% TIR between 63-140 mg/dL and < 4% TBR (12).

Scott and cols. conducted a multicenter study to evaluate the accuracy and safety of FreeStyle LibreTM, used to compare the control of 74 hyperglycemic pregnant women (T1DM, n = 24, T2DM, n = 11, and GDM, n = 39) with SMBG values. The results demonstrated good safety and acceptable reliability, compared to that reported in the non-pregnant population (30).

In another study, Sola-Gazagnes and cols. evaluated a discrepancy between isCGM/FGM results and SMBG based on the outcome of the study of 33 pregnant women. Although close, the results of the sensor underestimated those of the SMBG, in which 25% to 35% of management choices would have diverged if based on isCGM/FGM than SMBG (39).

Clinical studies have compared mostly pre-prandial capillary glucose measurements, which is a limitation. Despite recommendations for further studies, the use of isCGM/FGM can play an important role in improving glycemic control and the quality of life of pregnant women with hyperglycemia owing to its greater acceptability and simplicity compared to other CGM devices. However, the concomitant use of capillary glycemia should be recommended in association with isCGM/FGM for pregnant women with DM, mainly to confirm glucose values close to the lower limit (30).

Further studies should investigate whether improved glycemic control and pregnancy outcomes for the mother and baby can be achieved with prolonged use of isCGM/FGM during pregnancy (30).

## DIALYSIS

The prevalence of DM in recent decades is the main factor responsible for the substantial global increase in end-stage renal disease (ESRD). Currently, more than 3 million people worldwide are estimated to be receiving treatment for kidney failure, and the number is predicted to reach more than 5 million by 2035 (40). Chronic kidney disease, especially ESRD, causes disturbances of glucose homeostasis in subjects with and without diabetes (41). Since the kidney is a relevant site for glucose and insulin metabolism, glycemic control is particularly challenging in ESRD patients with diabetes, owing to a higher risk of hypo and hyperglycemia (42).

Although no evidence-based guideline is available for glycemic targets for hemodialyzed patients with T2DM, adequate glycemic control in those populations seems to be a predictor of survival (43). The Dialysis Outcomes and Practice Patterns Study included 9201 hemodialysis (HD) patients with T1DM or T2DM. Their results showed a U-shaped association between A1c and mortality, with the lowest mortality at A1c levels of 7% to 7.9%, thereby increasing progressively for either lower or higher A1c levels (44). Despite being challenging, adequate glycemic control to reduce glycemic variations should be targeted in this population (45).

In patients undergoing renal replacement therapy (RRT), the accuracy of A1c as a marker of glycemic control may be impaired by the altered half-life of red blood cells, erythropoietin therapy, anemia, uremic environment, and frequent blood transfusions, resulting in lowering A1c values (46). The latest Kidney Improving Global Outcomes guidelines highlight the inaccuracy of A1c, suggesting using continuous monitoring devices as an alternative (47).

CGM has emerged as a promising tool in glycemic control for patients with DM and undergoing the RRT. Although the available evidence about the management is currently scarce (48). In recent studies, CGM has proved useful in detecting asymptomatic hypoglycemia and glycemic variations, as well as in evaluating the effect of some medications in patients undergoing HD (49,50).

Since isCGM/FGM does not require calibration and has a longer useful life, it could be an adequate option for improving the quality of life and facilitating the treatment of DM individuals with ESRD. Flash glucose monitoring with FreeStyle Libre ProTM was shown to be acceptable by Yajima and cols. Parkes’ error grid analysis against SMBG showed that 49.0% and 51.0% of ISF glucose levels fell into zones A and B, respectively using isCGM/FGM. However, for isCGM/FGM, MARD against SMBG was significantly higher than that of CGM (19.5% vs. 8.1%, P <.0001) (51). Some studies reported similar results, revealing that isCGM/FGM average levels of 5 to 9.2 mg/dL were lower than the average of SMBG in patients with DM not undergoing HD (52,53). Possible explanations for the results were the effect of the edema in the arm and the abdomen, where sensors were placed to record isCGM/FGM and CGM, respectively. In addition, hematocrit may affect blood glucose meter performance in these patients, as low hematocrit values result in high readings.

Hissa and cols. conducted a 3-week prospective study to compare capillary and ISF glucose in patients with T2DM undergoing dialysis, which was measured by FreeStyle LibreTM. Results showed that MARD values were between 16.5 and 19.0% in the first week. In the second week, the MARD values ranged between 25.3 and 28,8%. Regarding the Clarke and Parkes error grid, 90.3% of patients were in Zone AB of Parkes and 89.6% of Clarke. The lower frequency in the AB zones could be partly because they used only measurements taken during dialysis, when there is manipulation of fluid volume and greater probability of finding discrepancies between capillary and ISF measurements (54).

Another study focusing on HD patients with T2DM revealed higher estimated A1c (eA1c) from using glycated albumin, BMI, and hemoglobin than eA1c using isCGM/FGM, particularly in patients with decreased BMI (55).

A pilot study of 10 patients undergoing HD and using FreeStyle Libre ProTM to record the presence of hypoglycemia revealed glucose levels of less than 70 mg/dL in 90% of patients. However, the glucose levels of 4 out of 10 were less than 55 mg/dL during the dialysis period. All episodes were asymptomatic, which is more dangerous and difficult to detect (56).

Mild or severe hypoglycemic episodes are known to be associated with an increased risk of cardiovascular events, hospitalization, and mortality. ESRD is often associated with neuropathy and impairing the perception of hypoglycemia in patients with diabetes. Although their risks can be reduced using fluid enriched with glucose, glycemic patterns are still difficult to predict. However, the application of isCGM/FGM could be helpful in this situation (56,57).

Additional studies are needed to determine the accuracy and safety of this form of blood glucose monitoring in patients with ESRD. Due to a lack of evidence at present, it is not recommended for people undergoing peritoneal dialysis. For those on HD, isCGM/FGM should be applied with caution. It is crucial to assess patterns rather than focusing on specific glucose values (58).

## INTENSIVE CARE UNIT

Dysglycemia, which includes stress-induced hyperglycemia, hypoglycemia, and excessive glycemic variability, is common amongst critically ill patients (59). Previous studies associate poor glycemic control with increased morbidity and mortality in critically ill patients (60). A recent meta-analysis demonstrated that intensive glucose control among critically ill patients reduces all-cause mortality. However, severe hypoglycemia is more frequent in this context, justifying continuous assessment of glycemic status (61). Currently, glucose control in the intensive care unit (ICU) is based on intermittent measurements using handheld meters for point-of-care testing. Handheld glucose meters are not designed for ICU settings, and their accuracy is questionable and markedly inferior to central laboratory or blood gas analysis, especially in patients with anemia or hypoxia, or those exposed to certain drugs (62). In critically ill patients, between 4% and 15% of hypoglycemic events are undetected, more frequently when there is a long time interval between glucose measurements (63).

Meanwhile, the CGM systems are gradually gaining space, as they can overcome these limitations and detect acute changes in glucose levels without overloading the nursing staff (64). However, some limitations of current CGM systems are remarked, originating from physiological and technical aspects. CGM systems’ technical performance and accuracy must be reliable to be used in daily practice. Data reliability about the patients with diabetes cannot be automatically transferred to a different situation like the ICU, where many variables (edema, hypotension, and vasoactive drugs, among others) can affect CGM performance (65).

A systematic review by van Steen and cols. identified 32 studies that addressed the accuracy of CGM. However, only five randomized controlled trials studies, focusing on the ICU population, explored the efficacy of CGM (63). Despite the conclusive evidence about the CGM failure to improve glycemic control, no formal meta-analysis could be conducted owing to a low number of studies, small sample size, group heterogeneity, and difference in glucose target values.

In addition, the literature is inconsistent regarding the detection of hypoglycemia by CGM. While Holzinger and cols. showed that CGM reduces episodes of hypoglycemia (66), Boom and cols. revealed no increase in the detection of hypoglycemic events with the system (64).

Ancona and cols. reported high reliability of the FreeStyle LibreTM system in eight adult patients admitted to the ICU; all had been diagnosed with DM. The authors observed acceptable values and clinical accuracy of arterial blood glucose, even higher than the capillary blood glucose levels (67).

Another study, evaluating the performance of isCGM/FGM in the pediatric ICU setting, did not show a satisfactory result, with a tendency of underestimating glucose levels. The differences in arterial blood gas, capillary blood, and biochemical serum are greater in the hypoglycemic and normoglycemic range than in the hyperglycemic range (68). The observation could explain the satisfactory results of the study by Ancona and cols., in which all patients had diabetes before hospitalization and, therefore, usually higher glucose values.

Zhang and cols. evaluated FreeStyle LibreTM feasibility and accuracy in 17 patients with COVID-19 and hyperglycemia admitted to the ICU. The error grid analyses against venous blood glucose showed acceptable clinical accuracy, with 97.1% of glucose readings falling into zones A and B in Clarke error grid analysis and 97.7% in zones A and B in consensus error grid analysis. However, MARD was 22.4%, which was higher than reported in outpatients with diabetes (69).

The lower values found with the isCGM/FGM may not reflect inaccuracy. However, it may reflect the difference in the glucose level measurement in plasma before glucose consumption by cells, as compared with interstitial after glucose utilization. Glucose diffusion is determined by blood supply, which may be impaired in patients with a critical illness, vasopressor therapy, or both. Simultaneously, exogenous insulin administration may augment glucose uptake through subcutaneous cells. Importantly, interstitial edema, common in critically ill patients, may further dilute the subcutaneous glucose and contribute to an increased glucose gradient between compartments (67).

Assessing the accuracy of subcutaneous and intravascular CGM devices is still a challenge, despite randomized clinical trials pointing to a lower accuracy of the subcutaneous device. However, the intravascular device requires an invasive procedure for placement, with an increased risk of peripheral venous thrombosis and infection. Schierenbeck and cols. showed a MARD of 30.5% versus 6.5% between FreeStyle LibreTM and Eirus (intravascular) after cardiac surgery (70). One of the hypotheses, although not reported by the authors, could be the possible reason for poor subcutaneous tissue perfusion, which was provoked by perioperative hypothermia, limiting its effectiveness (67).

Though a definitive conclusion has not been reached, the expert consensus is that CGM could offer improved glucose control with less risk of hypoglycemic events in ICU patients, justifying future research through a randomized controlled clinical trial (71).

## CIRRHOSIS

DM and glucose intolerance are observed in patients with liver cirrhosis since the liver plays a central role in glucose metabolism (72). A large cohort study showed that DM is an independent risk factor for developing chronic nonalcoholic liver disease, along with hepatocellular carcinoma (73). Furthermore, cirrhosis is a major cause of death in patients with DM (74).

In patients with cirrhosis, A1c generally underestimates glycemic status. Possible explanations include the shortened erythrocyte life span and anemia, frequently observed in patients with advanced liver disease, whether due to bleeding or hemolysis related to hypersplenism (75). Individuals with chronic liver disease have insulin resistance and hyperinsulinemia. Moreover, postprandial hyperglycemia is frequently observed in these patients. After an overnight fast, patients with cirrhosis will develop a metabolic profile similar to that found in normal individuals after two to three days of fasting, due to their low hepatic glycogen supply (76). SMBG provides information according to the recommended frequency, which usually occurs before and after meals. However, nocturnal hypoglycemia is not normally captured, and it is difficult to obtain information on fluctuations in glucose levels (77). Honda and cols. reported a direct relationship between a deterioration of the hepatic functional reserve and higher glycemic variability with CGM in individuals with DM. Their study identified hidden abnormalities of glucose fluctuations in this population, along with the presence of asymptomatic nocturnal hypoglycemia (78).

A recent study evaluated isCGM/FGM performance in patients with diabetes and liver cirrhosis (LC). Thirty-one patients in the study group and 30 controls with diabetes, but without liver disease, were analyzed. The results showed a strong agreement between isCGM/FGM readings and capillary glycemia (79). A MARD of 12.68% was found in the LC group, versus 10.55% in the control group, similar to the results of previous studies using different target populations (9,67,79). Nevertheless, MARD was persistently higher in those with LC as compared to the control, possibly due to the fluid overload characteristic of cirrhotic permanent hyperdynamic circulation and fluid retention state, significantly impacting isCGM/FGM's analytical accuracy. However, the clinical usability of isCGM/FGM in patients with LC was confirmed through Consensus Error Grid analysis, which resulted in 80.36% of values in zone A and 99.83% of values in zones A + B (79).

The systemic changes caused by liver dysfunction generate imprecision in markers used to monitor glycemic control. The reason is that in the initial stages, fasting blood glucose levels are normal in 23% of those with evident diabetes, revealing a proving difficult diagnosis (80).

Although the impact of early diagnosis and treatment of glycemic changes in patients with cirrhosis is unknown, it is tempting to speculate this as beneficial. While monitoring glycemic control in all DM stages, the isCGM/FGM could facilitate the monitoring and identification of glycemic variability and provide the appropriate therapy for identified changes. However, future studies are required to confirm the accuracy of the tool and its application for clinical practice (79).

## PHYSICAL EXERCISE

Physical activity (PA) plays important role in blood glucose management and the overall vitality of individuals with diabetes and prediabetes (81). Regular exercise can prevent or delay the development of T2DM. At the same time, it is part of an effective non-pharmacological intervention. Previous studies demonstrated that aerobic or combined (aerobic and resistance) exercise can reduce glucose variability in patients with diabetes (82,83). In addition, circulating markers of oxidative stress and inflammation, influenced by different exercise protocols, have been related to change in glucose variability in T2DM (84).

Moreover, physical exercises play vital roles in managing T1DM. In a large cross-sectional study of 18,028 adults with T1DM, patients who were most active in PA had better A1c levels, more favorable body mass indexes (BMIs), less dyslipidemia and hypertension, and fewer diabetes-related complications (retinopathy, microalbuminuria) than those who were less active (85).

Insulin action in muscle and liver can be modified by intensive exercise sessions and regular PA. Acute, aerobic exercise increases muscle glucose uptake up to fivefold through insulin-independent mechanisms. After exercise, glucose uptake remains elevated by insulin-independent (∼2 hours) and insulin-dependent (up to 48 hours) mechanisms if exercise is prolonged (86). Improvements in insulin action may last for 24 hours following shorter-duration activities (∼20 minutes) and if the intensity is elevated to near maximal effort (87). Even low-intensity aerobic exercise lasting 60 minutes or longer enhances insulin action in adults with obesity and insulin resistance for at least 24 hours (88).

In T1DM, blood glucose responses to PA are highly variable, considering the duration and intensity of the exercise, initial blood glucose levels, the individual anaerobic threshold, and the amount of insulin in circulation (81). In general, aerobic exercise decreases blood glucose levels if performed during postprandial periods along with the insulin dose administered at the meal before exercise, and prolonged activity may cause hypoglycemia (89,90). Exercise while fasting may produce a lesser decrease or a small increase in blood glucose (91). Anaerobic exercises and high-intensity interval training (HIIT) may provide better glucose stability, decrease blood glucose level, or slightly raise the glucose level (92,94). Due to greater glycemic instability that often requires therapy adjustments, a higher frequency of glucose monitoring is required in those who are physically active. A previous study identified hypoglycemia as the stronger barrier to regular PA in adults with T1DM (94).

Thus, the CGM appears to be a possible ally for this population, allowing frequent assessments of glucose levels more conveniently and painlessly. Information, obtained in real-time during and after PA, could help in insulin and carbohydrate intake adjustments during and after exercise, thereby reducing extreme fluctuations in blood glucose. However, recently, CGM devices have been extensively evaluated in the context of physical exercise due to the apparent delay between interstitial glucose readings and blood glucose in situations with rapid changes in concentrations (95).

A study evaluating the accuracy of CGM during prolonged aerobic exercise in patients with T1DM observed a delay of 12 ± 11 minutes between CGM and SMBG, in addition to an increase in MARD of 13% during the exercise (95). In line with this, Biagi and cols. reported an increase in MARD from 9.5% to 16.5% during aerobic exercise; whereas, the anaerobic exercise showed no significant difference (96).

In a previous study, the MARD for HIIT changed from 10.4% before exercise to 17.8% during training, in addition to a delay of 35 minutes in reaching half of the maximum glucose value compared to SMBG (97).

Aberer and cols. compared three different CGM devices – FreeStyle LibreTM (Abbott), Dexcom G4 PlatinumTM (Dexcom), and Medtronic MiniMed 640GTM (Medtronic) – during moderate aerobic activity, performed by individuals with T1DM, both before and after a meal. The outcome of the comparison reveals a high level of accuracy in all three devices during the exercise. The Abbott system was reported to have the best accuracy, with the lowest MARD (13.2 ± 10.9%) (98).

The fact that isCGM/FGM does not require calibration by the user and has longer sensor durability makes the system more convenient in different conditions of everyday life, including exercises (99). Giani and cols. assessed the performance of isCGM/FGM during an interval training exercise in young people with T1DM. The isCGM/FGM MARD during exercise was higher compared to the glucometer (12.5% vs. 5.7%). During the workout session, an increase in the MARD value was recorded from 5.5% at the beginning of the session to 15.8% at 30 minutes, suggesting a possible degradation of MARD during the training session, even if the variation was not statistically significant, probably due to the small sample size (99).

Another recent study evaluated the performance of FreeStyle LibreTM in T1DM patients during moderate aerobic exercise. Immediately before the exercise testing, isCGM/FGM performance in resting conditions showed an overall MARD of 13.7%; whereas overall MARD during exercise increased to 22% and reached 36.3% during exercise-induced hypoglycemia (100).

Zaharieva and cols. evaluated simultaneously two rt-CGM devices during aerobic exercise and one isCGM/FGM in a male patient with T1DM. They observed a significant delay of these devices concerning SMBG, with differences in measurements greater in the first 30 minutes of the exercise (101).

The CORRIDA study showed the superiority of rt-CGM to isCGM/FGM in reducing hypoglycemia and improving TIR in adults with T1DM and normal hypoglycemia awareness, demonstrating the value of rt-CGM alarms during exercise for daily diabetes self-management (102).

The performance of isCGM/FGM was assessed during the daily exercise and the challenges faced by individuals with T1DM. The overall MARD during inpatient phases was 14.3%. However, the overall MARD during acute exercise was 29.8%, not sufficiently accurate and required confirmatory blood glucose measurements (103).

Although isCGM/FGM is a potentially useful tool during and after PA, the lower performance of the system observed in a study requires additional confirmation of the results, especially in light of the change in the therapeutic protocol (100).

In conclusion, the development of an isCGM/FGM has transformed the management and treatment of patients with diabetes in recent years. Several clinical situations, previously presented difficulties for glucose monitoring, were contemplated in light of the benefits of the isCGM/FGM. Though current scientific evidence supports the use of the flash system in individuals with DM, future studies are required to reinforce its performance and safety.
